# FishMicrosat: a microsatellite database of commercially important fishes and shellfishes of the Indian subcontinent

**DOI:** 10.1186/1471-2164-14-630

**Published:** 2013-09-18

**Authors:** Naresh Sahebrao Nagpure, Iliyas Rashid, Rameshwar Pati, Ajey Kumar Pathak, Mahender Singh, Shri Prakash Singh, Uttam Kumar Sarkar

**Affiliations:** 1 Division of Molecular Biology and Biotechnology, National Bureau of Fish Genetic Resources, Lucknow 226002, India; 2 Division of Fish Taxonomy and Resources, National Bureau of Fish Genetic Resources, Lucknow 226002, India

**Keywords:** Microsatellite loci, Tandem repeats, Polymorphism, Primer, Flanking region, Fish

## Abstract

**Background:**

Microsatellite DNA is one of many powerful genetic markers used for the construction of genetic linkage maps and the study of population genetics. The biological databases in public domain hold vast numbers of microsatellite sequences for many organisms including fishes. The microsatellite data available in these data sources were extracted and managed into a database that facilitates sequences analysis and browsing relevant information. The system also helps to design primer sequences for flanking regions of repeat loci for PCR identification of polymorphism within populations.

**Description:**

FishMicrosat is a database of microsatellite sequences of fishes and shellfishes that includes important aquaculture species such as *Lates calcarifer*, *Ctenopharyngodon idella*, *Hypophthalmichthys molitrix, Penaeus monodon*, *Labeo rohita*, *Oreochromis niloticus*, *Fenneropenaeus indicus* and *Macrobrachium rosenbergii*. The database contains 4398 microsatellite sequences of 41 species belonging to 15 families from the Indian subcontinent. GenBank of NCBI was used as a prime data source for developing the database. The database presents information about simple and compound microsatellites, their clusters and locus orientation within sequences. The database has been integrated with different tools in a web interface such as primer designing, locus finding, mapping repeats, detecting similarities among sequences across species, and searching using motifs and keywords. In addition, the database has the ability to browse information on the top 10 families and the top 10 species, through record overview.

**Conclusions:**

FishMicrosat database is a useful resource for fish and shellfish microsatellite analyses and locus identification across species, which has important applications in population genetics, evolutionary studies and genetic relatedness among species. The database can be expanded further to include the microsatellite data of fishes and shellfishes from other regions and available information on genome sequencing project of species of aquaculture importance.

## Background

Microsatellites are observed in almost all known eukaryotic and prokaryotic genomes, present in both coding and non-coding regions. They have a high mutation rate (between 10^-3^ and 10^-4^ mutations per gamete per generation) that generates and maintains extensive length polymorphism [1,2]. This makes microsatellite a powerful genetic marker for a variety of applications like population genetics, genetic linkage mapping, parentage assignment, marker assisted selection, molecular breeding, and allele mining [3,4]. A microsatellite locus generally varies in length between 5 to 40 repeats. Di-, tri- and tetranucleotide repeats are the most common choices for molecular genetic studies. Dinucleotides are an abundant type of microsatellite repeat found in most vertebrates, whereas trinucleotide repeats are most abundant in plants [5-7]. Microsatellites represent ideal molecular markers because they have multiple alleles that are highly polymorphic among individuals and loci that are highly abundant and dispersed evenly throughout eukaryotic genomes. The major drawback of using microsatellite is that for most species they need to be developed *de novo*, a process that is often costly and protracted [8]. Efforts have been made worldwide to compile and develop online and offline microsatellite databases of biological organisms [9-15]. Valuable studies have been done in fishes such as microsatellite genetic linkage maps [16-20], characterization and identification of microsatellites [21-24] and cross-species microsatellite locus identification [25-27]. Despite the importance of microsatellite markers, meagre efforts have been made to develop a microsatellite database of the fishes except *Danio rerio*[28], *Cyprinus carpio*[29] and Fishgen [30].

In this article, we describe the development of a microsatellite database (FishMicrosat) for population genetics and stock management using LAMPP (Linux-Apache-MySQL-PHP-Perl) technology and GenBank of NCBI as a data source to extract the microsatellite data. FishMicrosat is a unique database of microsatellite sequences that covers commercially important fish and shellfish species of the Indian subcontinent. The database currently contains 4398 sequences of 41 species belonging to 15 families and provides information on the type of repeat in terms of mono-, di-, tri-, tetra-, penta and hexanucleotide, simple and compound microsatellite, along with the characteristic of repeats namely size, region, pattern & unit. Additionally, algorithms were implemented for finding loci across species, based on the presence of identical simple sequence repeats (SSRs) with the same or varying frequencies of repeat units but conserved flanking regions. The database is regularly updated based on the release of new records in GenBank for the existing 41 species as well as the addition of new species belonging to the Indian sub continent. It is expected that the database will be a valuable resource in many aspects of fish genetic research of the Indo-Pacific region, Bay of Bengal and Arabian Sea.

## Construction and content

### Data source

Microsatellite sequences of fish and shellfish species were downloaded from Entrez of NCBI [31] using the keyword search ‘Fish microsatellite’ under nucleotide. Files were downloaded in GenBank and FASTA format for annotation and sequences respectively. Further, a Perl program (SpciesExtractor.pl) was written and used for data extraction for only important species found in the Indian subcontinent from the downloaded files. Other physical information about the species like habitat, distribution, IUCN Red List status was collected from FishBase [32]. Another Perl parsing program (InformationParser.pl) was developed to extract the information from the files according to the database schema and manage the data into the database. These Perl programs are used by the database administrator for updating based on new releases of microsatellite sequences for existing and new species.

### Design and development

#### Database

**Figure 1:**
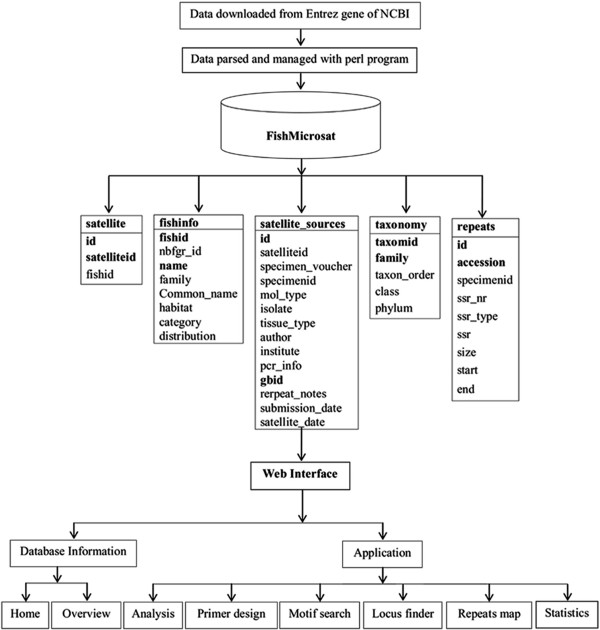
Architecture and data flow representation in ‘FishMicrosat’.

In order to manage the data, MySQL, a relational database management system, was used for building the database. Tables were designed and relationships among tables were created using unique, primary and foreign keys. Five tables were designed to store the information about microsatellite sequences and species. Table ‘fishinfo’ contains the physical and phenotypical information; ‘satellite_sources’ holds details molecular information about microsatellites; ‘satellite’ works as a bridge between tables ‘fishinfo’ and ‘satellite_sources’; ‘taxonomy’ shows systematic information of the species and acts as a sub table of ‘fishinfo’. And finally the table ‘repeats’ covers the data about repeats of all microsatellites sequences obtained by using the repeat analysis program ‘MISA’ [33] as shown in Figure 1.

#### Web interface

A web interface integrated with the database was designed and developed to retrieve and access the information of interest using web technologies like PHP, HTML, CSS, JavaScripts, DBI (Database Interface), CGI (Common Gateway Interface), GD (Graphic Design) and Perl. The web interface also incorporates the different tools for searching, viewing and analysing the microsatellite data (Figure 1).

#### Identification of microsatellite loci across species

**Figure 2:**
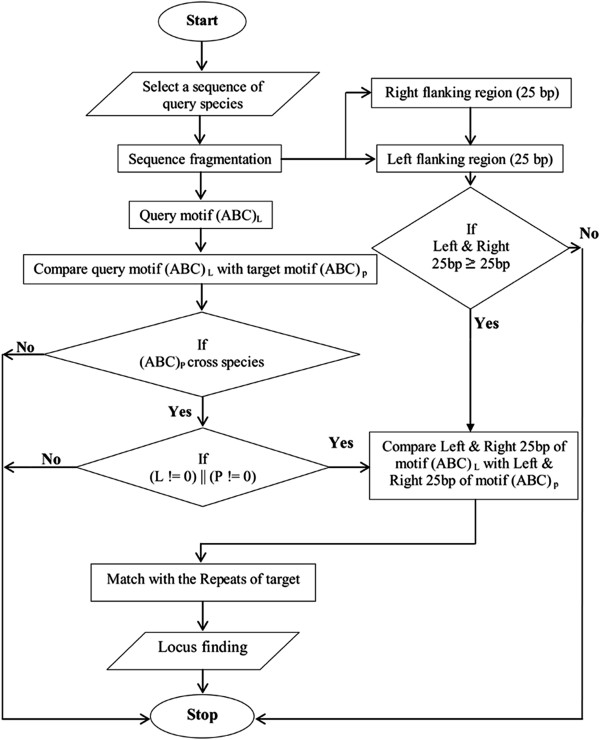
Flowchart of the locus finder algorithm.

The microsatellite loci among the existing sequences were identified by implementing an algorithm into a program (locusfinder.pl) using Perl. In order to construct the algorithm, a microsatellite sequence of selected species was divided in parts (a) motif of repeats region (b) 25 bp flanking sequence upstream and (c) 25 bp flanking sequence downstream to the repeat region. The repeat region and motifs present in sequences of selected species were fetched from the ‘repeats’ table of the database sequentially for retrieving identical target motifs and its sequences. Further, the conserved flanking regions were checked in query as well as target sequences. The evolutionary conservation of the flanking region allows hetero specific identification of SSRs [34]. These conserved flanking regions have been used for designing PCR primers for microsatellite amplification and genotyping of individuals of the same species as well as across species [35,36]. Thus, to identify loci across species, an algorithm was designed by considering the approach for example ‘ABC’ as a repeat pattern and ‘L’ the number of repeat units in a selected query sequence. The same repeat pattern ‘ABC’ was used to check its availability and repeat frequency (denoted as P) in the target sequence (Figure 2). Here, because the repeat frequency may be polymorphic, the value of the repeat frequency in the selected query sequence (L) may or may not be equal to the repeat frequency in the target sequence (P) i.e. L = P or L! = P. The algorithm uses a 25 bp length of flanking region on either side, which is sufficient for amplification of a microsatellite locus in a PCR reaction for laboratory validation. The loci identification program supports the findings of the previous studies that microsatellite repeats vary within and between different genomes of organisms [37,38].

#### Search and analysis

Apart from locus finding across species, other search and analysis modules like ‘Keyword search’, ‘Repeat analysis and primer’, ‘Motifs search’, and ‘Repeats map’ were implemented and integrated in the web interface for browsing information. The ‘Keyword search’ takes a word like ‘fish name’, ‘common name’, ‘family’, ‘accession number’ and ‘author’ typed in by the user as input and performs the search. The ‘Motif search’ takes input parameters like motifs, repeat length and repeat type (simple or composite) and returns the result with the help of regular expression programming and SQL (Structured Query Language) concepts. The ‘Repeats map’ was developed using Perl, MISA and Blastn [39] programs for identifying similarities among the sequences and mapping the repeats. The Blastn program uses ‘blastmsdb’ database, which is a blast compatible and created apart from the main database by using ‘formatdb’ program of blast package. ‘Repeats map’ analyzes and process the input query sequence through the MISA program to generate the repeats. If repeats are found, it further leads to alignment with other similar sequences; otherwise the program terminates with a warning message. Primer3 program [40,41] was used for primer designing, and a standalone version was downloaded [42] to compute multiple sets of forward and reverse primers for microsatellite loci along with melting temperature (Tm), GC content, start position and product size. These generated primers can be used in PCR reactions for identification of polymorphic loci for genotyping of individuals.

#### Implementation of ‘statistics’

The MISA program was implemented to ascertain the frequently occurring repeat types and repeat information from all the sequences in FishMicrosat. The results obtained from MISA were parsed and stored in the ‘repeats’ table of the database. The ‘GD graph’ module was used to design and dynamically display the frequency of different types of repeats (mono to hexa) in the ‘Pie diagram’. The ‘Pie diagram’ presents the frequency of each type of repeat and is revised when the database is updated.

## Discussion and utility

FishMicrosat covers 4398 microsatellite records of 41 commercially important aquaculture species belonging to 15 families (Table 1). The Home page of the web interface of FishMicrosat integrated with different analytical modules, presents the numerical statistics of the top 10 families and species in addition to providing information on updating and current status of the database. The ‘Top 10 FishMicrosat families’ displays ten families which has the largest number of species in FishMicrosat and the ‘Top 10 FishMicrosat Species’ displays the ten species for which the largest number of specimen records are available in the database. The page also provides an overview of FishMicrosat and its features. The analytical tools like motif, sequence similarity search and repeat mapping, and finding microsatellite loci across species were integrated to increase the utility and scope of the database.

**Table 1:** Distribution of SSR’s by species

**S no.**	**Species**	**Simple**							**Compound**
		**Sequences**	**Mono**	**Di**	**Tri**	**Tetra**	**Penta**	**Hexa**	
1	*Amphiprion sebae*	1	0	1	0	0	0	0	0
2	*Catla catla*	22	1	25	1	4	0	0	0
3	*Channa marulius*	2	0	2	0	0	0	0	0
4	*Chitala chitala*	31	0	31	1	6	0	0	4
5	*Cirrhinus cirrhosus*	7	0	6	0	0	0	0	0
6	*Cirrhinus mrigala*	11	0	9	1	1	0	0	0
7	*Ctenopharyngodon idella*	500	18	523	29	70	0	0	31
8	*Epinephelus fuscoguttatus*	312	43	167	29	11	2	0	5
9	*Fenneropenaeus indicus*	182	11	96	26	86	1	0	18
10	*Garra gotyla*	26	2	13	0	0	0	0	1
11	*Heteropneustes fossilis*	10	5	11	0	0	0	0	0
12	*Hippocampus kuda*	12	1	10	1	2	0	0	0
13	*Hippocampus trimaculatus*	12	1	12	1	2	0	0	0
14	*Horabagrus brachysoma*	11	0	12	2	0	0	0	0
15	*Hypophthalmichthys molitrix*	402	42	425	18	59	1	0	27
16	*Hypselobarbus curmuca*	8	1	7	2	1	0	0	0
17	*Katsuwonus pelamis*	5	1	7	1	0	0	0	0
18	*Labeo bata*	4	0	4	0	0	0	0	0
19	*Labeo calbasu*	10	0	8	0	1	0	0	1
20	*Labeo dussumieri*	7	0	2	2	1	0	0	1
21	*Labeo dyocheilus*	10	0	6	0	1	0	0	1
22	*Labeo fimbriatus*	17	1	28	0	0	0	0	0
23	*Labeo pangusia*	11	0	10	0	0	0	0	1
24	*Labeo rohita*	531	9	689	169	38	0	5	26
25	*Lates calcarifer*	975	43	903	53	157	8	0	51
26	*Macrobrachium malcolmsonii*	5	0	5	0	0	0	0	2
27	*Macrobrachium rosenbergii*	180	20	151	106	3	0	0	16
28	*Mystus oculatus*	2	0	1	0	0	0	0	1
29	*Mystus tengara*	1	0	1	0	0	0	0	0
30	*Notopterus notopterus*	8	0	13	0	1	0	0	0
31	*Oreochromis niloticus*	303	14	399	7	2	0	0	11
32	*Osteobrama belangeri*	3	0	2	0	0	0	0	0
33	*Pampus argenteus*	31	3	45	0	3	0	0	2
34	*Pangasius pangasius*	28	1	37	2	0	0	0	1
35	*Penaeus monodon*	618	59	366	155	104	3	6	68
36	*Puntius chalakkudiensis*	11	0	21	1	0	0	0	1
37	*Puntius denisonii*	11	0	17	0	0	0	0	0
38	*Rita gogra*	2	0	3	0	0	0	0	0
39	*Schizothorax richardsonii*	53	1	119	3	0	0	0	7
40	*Sinilabeo dero*	20	0	15	0	1	0	0	3
41	*Sperata aor*	3	0	5	0	0	0	0	0
**Total**	**41**	**4398**	**277**	**4207**	**610**	**554**	**15**	**11**	**279**

### Browsing specimen information

The specimen records of the species of interest can be viewed by using the species instantiation index under the ‘Record overview’ menu item in the web interface. Hyperlinked navigational indexes by first letter of the generic name have been provided to find the species of interest along with the number of specimen records in square brackets. Further, selection of each species name is hyperlinked and a mouse click over the species name presents information on family, common name, habitat, distribution, microsatellite repeats, its region and size, sequence length, authors and NCBI accession number. The NCBI accession number for each specimen record also has a hyperlink to NCBI. The ‘Top 10 FishMicrosat families’ and ‘Top 10 FishMicrosat Species’ on the home page of the web interface provides other means of viewing information about the species and its specimens.

### Keyword search

**Figure 3:**
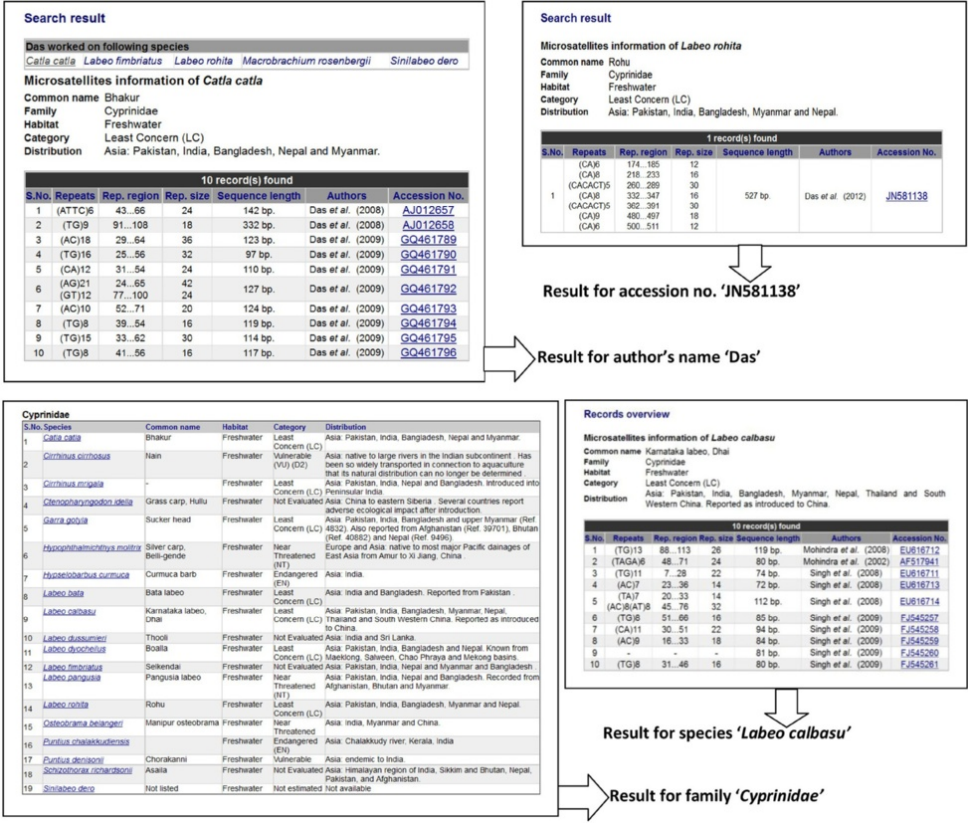
Search results for different keywords in ‘FishMicrosat’.

The keyword search works on keywords like species name, common name, family name, accession number, and author for retrieving the information from the database. Different views have been created for all these keywords to present relevant information from the database. For example the species name or common name as an input keyword leads to record overview. The ‘author view’ displays a list of all the species on which the particular author worked and also displays the specimen records which corresponds to the listed species. Similarly, family name and accession number keywords also lead to respective views (Figure 3).

### Repeat analysis and primer design

**Figure 4:**
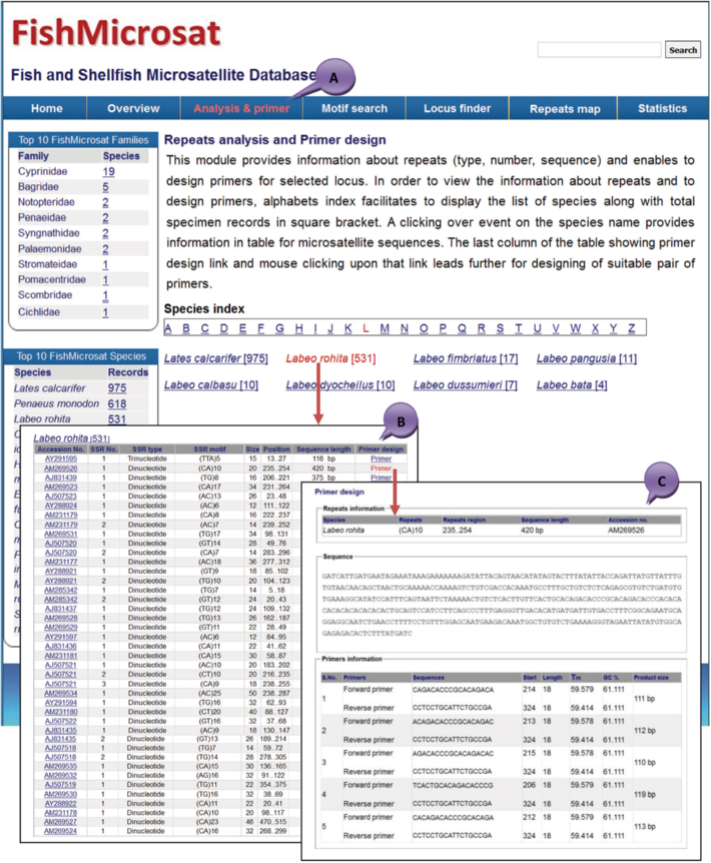
**The web layout for SSR analysis and primer design of ‘FishMicrosat’. (A)** Species specific SSR analysis and primer design **(B)** repeat analysis output **(C)** SSR specific primer design output.

The menu item ‘Analysis & primer’ (Figure 4A) detects repeats in the sequences and designs the primer for the selected repeat locus. Thus, to obtain the repeat information and design primers for a specific repeat, the end user selects a species of interest starting with a generic name. Clicking on the species name provides a table that contains information such as accession no., SSR no., SSR type, SSR motif, SSR size, position, sequence length and a link for primer design for each specimen (Figure 4B). For primer designing, the ‘Primer3’ standalone program computes primers upon user request for microsatellite sequences that have suitable length of flanking regions and ample GC content in that region; otherwise the request is rejected with a warning message (Figure 4C). The program displays a list of multiple primers along with respective values for Tm, GC content, start position and product size (oligo size). The primer sequences will be useful in determining the alleles and finding of loci across species.

### Motif search

**Figure 5:**
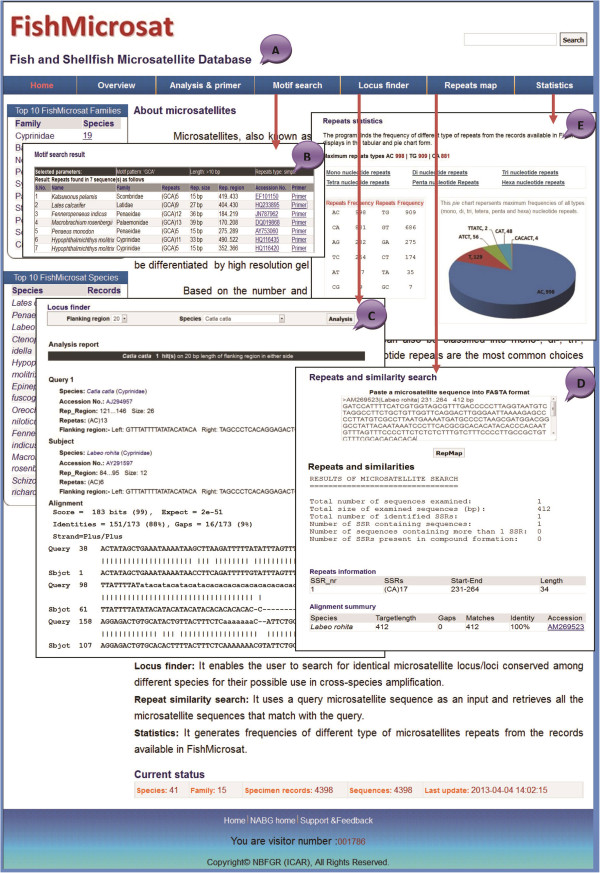
**The different web layouts of ‘FishMicrosat’ for data retrieval and analyses. (A)** Home page **(B)** Motif search **(C)** Locus finder **(D)** Repeats map **(E)** Repeats statistics.

A repeat motif can be searched from the menu item ‘Motif search’ integrated in the web interface (Figure 5A). It searches repeats in all microsatellite sequences present in the database and fetches information on species name, family, repeats, size, repeat region, NCBI references, and primers for SSRs (Figure 5B). Three input values are required under ‘Motif search’: ‘Motif’ for nucleotide pattern (mono-hexa), ‘Length’ for number of nucleotides (i.e. > 10) and ‘Repeats type’ (simple or compound). The search results provide a primer link that leads to the design of the primer for the corresponding repeat type.

### Locus finder

The Locus finder tab accesses identical microsatellite loci across species based on conserved flanking sequences (approximately 25 bp long) on either side of the polymorphic loci. The program uses two input parameters; length of flanking region and species name. Finding the identical microsatellite locus in other species existing in the FishMicrosat database, is highly useful for cross species amplification of microsatellite loci. For example the sequence of *Labeo rohita* (GenBank accession no. AY291597) and *Catla catla* (GenBank accession no. AJ294957) contain the same motif with conserved flanking regions on the parameter: flanking region ‘20 bp’ and species ‘*Labeo rohita*’. The sequence alignments are 88% identitical, indicating homology between sequences (Figure 5C).

### Repeats map

Repeat mapping and sequence similarity searching can be achieved through the menu item ‘Repeats map’ included in the web interface. The program accepts microsatellite sequences in FASTA format as input in the provided text area. The output presents information on repeats (size of query sequence, presence of compound/composite repeats, number of identified repeats in query sequence SSRs, SSRs number, repeats location and size of repeats) along with summary on alignment of identical/similar sequences. The alignment summary presents targeted sequences accession no., species name, target length, gaps, matches and identity between query and targeted sequences (Figure 5D). The program initially checks the presence or absence of the repeats in the input sequence and assigns a boolean value. If the value is true the program processes the query sequences by using Blastn program and its compatible ‘blastmsdb’ database for similar sequence searches. Thus, it helps to find information about repeats orientation and sequence similarity for the newly generated microsatellite sequences.

### Repeat statistics

In order to determine the frequency of different types of repeats from the specimen records available in FishMicrosat, the menu item ‘Statistics’ generates the frequency of each motif found in the database and displays the top three (most common) motifs with the largest frequencies. For example, the statistic view shows that repeats ‘AC’ was found 998 times, ‘TG’ 909 and ‘CA’ 881 throughout all sequences. A repeat type index has also been included to display all the repeats and their frequencies in a table. The dinucleotide repeat type selected as default displays 12 combinations of dinucleotides. The maximum frequency of each type of nucleotide repeats (mono to hexa) can be viewed in the pie diagram (Figure 5E). The largest frequency for a mononucleotide repeat is ‘T’ with 129 occurrences, dinucleotide repeats ‘AC’ with 998 occurrences, trinucleotide repeats ‘CAT’ with 48 occurrences, tetranucleotide ‘ATCT’ with 56 occurrences, pentanucleotide ‘TTATC’ with 2 occurrences and hexanucleotide ‘CACACT’ with 4 occurrences. The database with 4398 sequences of 41 species has 277 mono, 4207 di, 610 tri, 554 tetra, 15 penta, 11 hexa and 279 compound repeats (Table 1). This section also analyzes information on the occurrence of the most frequent and rare nucleotide repeats in the fish genome. The dinucleotide repeats AC|TG (998|909) and CA|GT (881|686) were frequently found while CG|GC (9|7) were rare in fish genome.

## Conclusions

FishMicrosat is a database of microsatellite sequences of commercially important fishes including shrimps and currently covers 4398 specimen records for 41 species. The database facilitates mining of SSR motifs, repeat orientations and sequence similarities. The statistics presents the relative abundance of microsatellite repeats that occur frequently in the genomes. Additionally, it facilitates in identifying polymorphic loci across species and designing primers for repeat loci, thus providing researchers ready to use information from a centralized location, avoiding the cumbersome process of referring to multiple sources of literature and using multiple programmes. This repository with included tools can play a key role in cutting edge areas of research by assisting with marker selection, linkage mapping, population genetics, evolutionary studies, genetic relatedness among the species and genetic improvement programmes of important aquaculture species.

## Availability and requirement

FishMicrosat is freely accessible at URL http://mail.nbfgr.res.in/fishmicrosat/ for research and academic use.

## Competing interests

The authors declare that they have no competing interests.

## Authors’ contributions

NSN and MS conceived this study. IR, RP & AKP created the work-flow, database, application modules and performed data analyses. SPS and UKS incorporated general information and taxonomy of fishes. IR, RP, NSN, AKP and MS drafted the manuscript. All authors read and approved the final manuscript.
